# Efficacy of Portal Vein Embolization with a Procedure of Sheath Injection and Balloon Occlusion with Gelatin Sponge

**DOI:** 10.5334/jbsr.2485

**Published:** 2021-09-09

**Authors:** Yosuke Nozawa, Hirokazu Ashida, Kenkichi Michimoto, Shunsuke Kisaki, Rui Kano, Hiroya Ojiri, Toru Ikegami

**Affiliations:** 1 Jikei university, JP

**Keywords:** future liver remnant, gelatin sponge, hepatic resection, portal vein embolization, balloon occlusion

## Abstract

**Introduction:**

To evaluate the efficacy, safety, and associated complications of a novel and simple approach to portal vein embolization that utilizes sheath injection and balloon occlusion (PVE-SIBO) with gelatin sponge (GS) for the purpose of increasing future liver remnant (FLR) volume.

**Methods:**

Between 1 January, 2006, and 31 August, 2020, 20 patients (15 men, 5 women, aged 64.6 ± 10.2 years) diagnosed with hepatobiliary malignancy underwent presurgical PVE-SIBO at our institution via a percutaneous transhepatic approach to the right portal vein and embolization of the portal vein with GS. We evaluated the increased ratio of FLR volume, operation duration, recanalization rate, and complications following this procedure.

**Results:**

All procedures were successful and without complications such as subcapsular hematoma, intra-abdominal bleeding, and bile leakage. The increased ratio of FLR volume was 34.7 ± 23.7% after a mean of 14.3 ± 2.57 days, and there was a significant difference in the FLR volume before and after PVE (*P* < 0.01). Procedure time was 52.7 ± 11.4 minutes.

**Conclusion:**

PVE-SIBO with GS is a simple, effective, and safe procedure to increase the ratio of FLR volume prior to hepatic surgeries.

## Introduction

Portal vein embolization (PVE) is an established presurgical procedure intended to increase the volume and function of the future liver remnant (FLR) prior to hepatic resection and thereby decrease the risk of associated morbidity and mortality [1,2]. Many reports have described the efficacy and safety of PVE using various institution-dependent PVE techniques and embolic materials, such as absolute ethanol, n-butyl-2-cyanoacrylate (NBCA), ethanolamine oleate (EOI), coil, polyvinyl alcohol (PVA), and gelatin sponge (GS) [3]. Nevertheless, these methods and materials have their limitations. One approach often used to perform PVE involves a reverse-curve catheter technique, but this method may be time-consuming according to portal vein anatomy and it is sometimes difficult to obtain the required shape. Similarly, a nonabsorbable agent is commonly used to carry out permanent vascular occlusion [4], but a temporary embolization agent is often used because it’s easy to use [5]. Performed prior to hepatectomy, PVE should be as simple and safe as possible. Accordingly, our protocol has been to inject GS via a gap of sheath and straight catheter under proximal balloon occlusion (PVE-SIBO). We believe few reports describe the use, safety, efficacy, and associated complications of this method; therefore, we undertook a retrospective evaluation of these considerations in our patients.

## Materials and Methods

Our institutional review board approved this retrospective study and waived the requirement for patients’ informed consent for the use of their data for this study.

### Patients

We reviewed 26 cases where PVE was performed prior to hepatectomy between 1 January, 2006, and 31 August, 2020, at our institution, and 20 of these patients (15 men, 5 women, aged 64.6 ± 10.2 years) met the criteria of our study. Inclusion criteria comprised of right portal vein puncture and embolization of the right portal vein using PVE-SIBO with GS alone. Six patients were excluded from this study according to the following exclusion criteria: embolization with GS and absolute ethanol (n = 2); left portal vein puncture (n = 3); PVE using reverse-curve catheter technique (n = 1).

Among the 20 patients, four patients had chronic liver disease secondary to alcoholic hepatitis, hepatitis B, or hepatitis C. One patient had received transcatheter arterial chemoembolization using farmorubicin prior to PVE-SIBO. Hepatic functions of all patients were assessed with Child-Pugh score system. Child-Pugh scores of the included patients were A (n = 17) and B (n = 3) before intervention (***Table 1***).

**Table 1:** Clinical characteristics of 20 patients.


CASE	AGE/SEX	DISEASE	AETIOLOGY	CHILD-PUGH CLASSIFICATION	CHEMOTHERAPY BEFORE PVE

1	71/M	CCA	Normal	B	None

2	68/F	HCC	Normal	A	None

3	75/F	CCA	Normal	A	None

4	60/M	MLC	Normal	A	None

5	74/M	HCC	Hepatitis B virus	A	TACE

6	60/M	MLC	Normal	A	None

7	67/M	CCA	Normal	A	None

8	71/M	MLC	Hepatitis B virus	A	None

9	72/M	GBC	Normal	A	None

10	43/F	MLC	Normal	A	None

11	76/M	CCA	Normal	A	None

12	50/F	CCA	Normal	A	None

13	61/M	HCC	Normal	A	None

14	61/M	HCC	Normal	A	None

15	65/M	MLC	Normal	A	None

16	81/M	CCA	Normal	B	None

17	74/M	MLC	Alcoholic hepatitis	A	None

18	63/M	CCA	Normal	A	None

19	50/F	MLC	Hepatitis C virus	A	None

20	50/M	CCA	Normal	B	None


CCA: cholangiocarcinoma; GBC: gallbladder cancer; HCC: hepatocellular carcinoma; MLC: metastatic liver cancer; TACE: transcatheter arterial chemoembolization: PVE: portal vein embolization.

All PVE procedures were requested to reduce the risk of postoperative hepatic failure. A hepatic surgeon and interventional radiologist discussed the indication. A preprocedural ratio of FLR to total liver volume (TLV) not exceeding 45% was required for all cases.

The preoperative diagnoses of the 20 patients were biliary carcinoma (n = 8), liver metastases (n = 7), hepatocellular carcinoma (n = 4), and gallbladder carcinoma (n = 1). The portal vein anatomies of the patients were normal (A status) according to Nakamura classification [6].

## PVE-SIBO procedure

**Figure 1:**
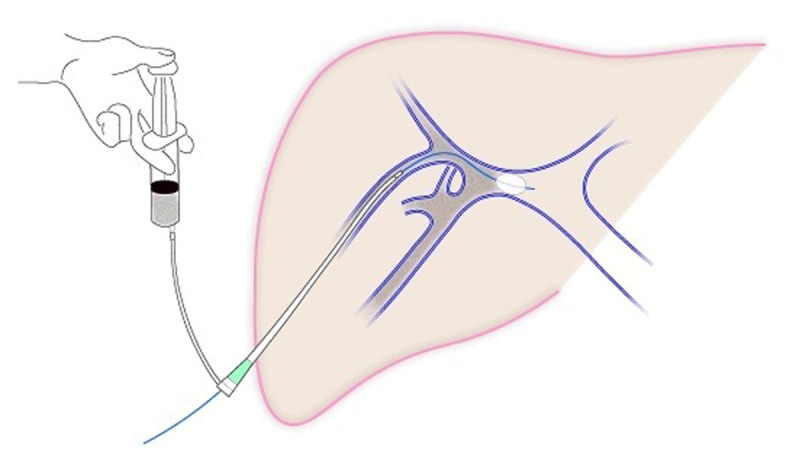
Scheme depicting the technique of portal vein embolization using sheath injection and balloon occlusion with gelatin sponge.

**Figure 2:**
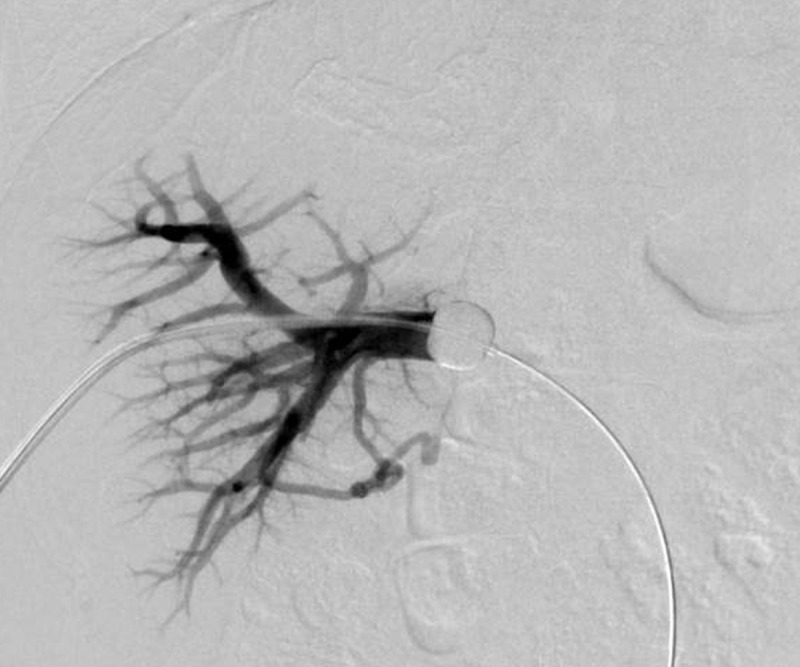
Portography of a 50-year-old woman with metastatic liver cancer just before portal vein embolization.

**Figure 3:**
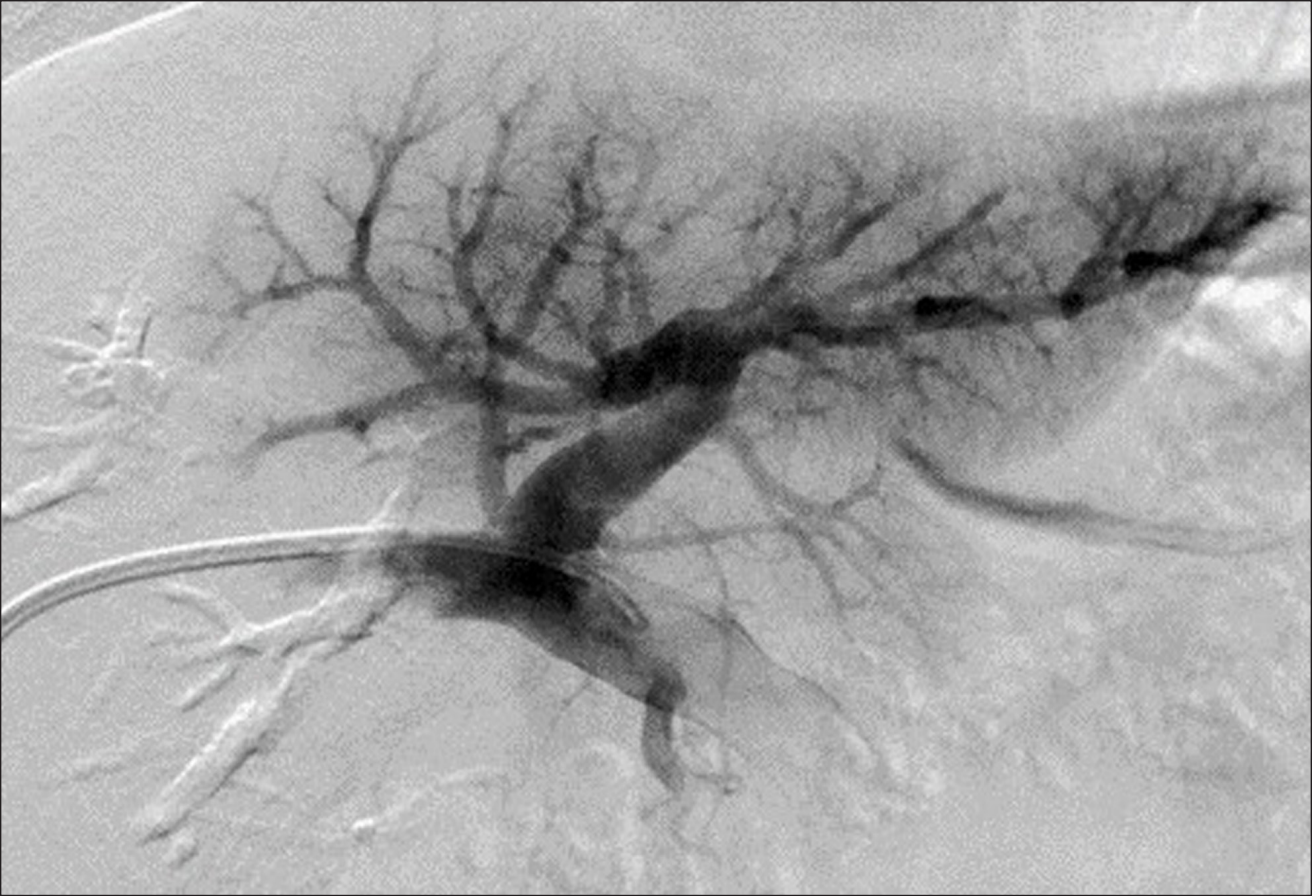
Portography shows complete obstruction of the right portal vein after embolization.

After local anaesthesia, a percutaneous transhepatic approach to the right portal vein branch was performed. A 6- to 8-French sheath (MEDIKIT catheter introducer; Togo Medikit, Tokyo, Japan) was inserted, a balloon catheter (Selecon MP catheter II, 9- to 20-mm diameter; Terumo Clinical Supply, Gifu, Japan; Moiyan 5-Fr, 9-mm balloon; Tokai Medical Products, Inc., Aichi, Japan; or Occlusion catheter, 5-Fr, 20-mm balloon; Nipro, Osaka, Japan) was inflated to occlude the proximal region of the right portal vein, and a mixture of one sheet of 1-mm square sized gelatin sponge (GELFOAM™, Pfizer, Tokyo, Japan) pieces and 10-ml of contrast agent was injected into the vein through the gap between the sheath and the balloon catheter (***Figure 1***). Portography was performed to confirm occlusion of the vein via the balloon catheter (***Figures 2*** and ***3***). Fibered coils (Tornado, 3 mm × 5 cm, COOK Medical, Tokyo, Japan) were used to embolize the PVE tract, and the sheath was removed.

### Evaluation

Technical success was defined as complete obstruction of the right portal vein on digital subtraction portography and procedure time was defined as the interval from insertion of sheath to the right portal vein to confirmation of its occlusion by portography. The time to refine GS prior to embolization was included in the procedure time.

### Analysis of efficacy, safety, and complications

Acquisition of arterial, portal, liver venous, and equilibrium phases of enhanced computed tomography (CT) were obtained before and after PVE. FLR volume and TLV were calculated using CT volumetric assessment software (Organs Volume Analysis, Hitachi Medico, Chiba, Japan or Volume Analyzer SYNAPSE VINCENT, FUJIFILM, Tokyo, Japan) on CT images with a slice thickness of 2 mm. We obtained the ratios of FLR/TLV before and after PVE and defined increased ratio as: Increased ratio = FLR volume after PVE – FLR volume before PVE/FLR volume before PVE × 100.

Complications and recanalization of the right portal vein were also radiologically evaluated on CT after PVE. Complications were categorized using the Clavien-Dindo classification system [7], and those classified above Grade 3 were defined as significant.

### Statistical analysis

We analysed statistics using R version 3.6.0 (R Foundation for Statistical Computing, Vienna, Austria) and compared data using the paired t-test. A *P* value below 0.05 was considered statistically significant.

## Results

PVE-SIBO was performed successfully in all 20 cases, with complete obstruction of the right portal vein as confirmed on digital subtraction portography at the end of the procedure. GS was the only embolization agent used. The mean amount of GS sheets and contrast media required per one session were 5.87 ± 3.98 sheets and 58.7 ± 39.8 ml, respectively. Though the mixture of GS used in our study was slightly viscous, it was smoothly injected through the gap between the sheath and catheter in the PVE-SIBO procedure. Sheath size varied from 6- to 8-French depending on the type of balloon catheter, but this caused no difficulty in injection. Procedure time for all but Cases 1 and 2, for which information was insufficient, was 52.7 ± 11.4 minutes.

CT scanning performed after a mean of 14.3 ± 2.57 days after PVE did not show any PVE-related complications above Grade 3, including subcapsular hematoma, intra-abdominal bleeding, or bile leakage. ***Table 2*** allows comparison of the FLR volumes before and after initial PVE. The FLR volume increased from 413.5 ± 133.0 mL to 556.3 ± 190.1 mL. The increased ratio of FLR volume was 34.7 ± 23.7% and there was a significant difference in the FLR volume before and after initial PVE (*P* < 0.01).

**Table 2:** Liver volume data before and after initial session of PVE-SIBO using GS.


CASE	CT SCAN AFTER PVE, DAYS	FLR BEFORE PVE, ML	FLR AFTER PVE, ML	FLR/TLV RATIO, % BEFORE PVE	FLR/TLV RATIO, % AFTER PVE	INCREASE RATIO, %	PROCEDURE TIME, MINUTES	CLINICAL OUTCOME

1	13	333	450	38.2	49.8	35.1		ERH

2	11	235	240	18.3	20.1	2.1		ERH

3	12	274	352	28.6	34.8	28.5	47	Inoperable

4	12	465	535	29.6	33.7	15.1	61	ERH

5	13	484	569	41.7	49	17.6	74	ERH

6	13	502	733	33.2	50.1	46.0	52	ERH

7	14	468	684	32.1	46.7	46.1	40	Inoperable

8	15	329	421	22.1	28.3	27.9	56	ERH

9	12	422	618	26.1	34.3	46.4	42	ERH

10	14	490	663	34.7	47	35.3	66	Inoperable

11	20	436	805	33.3	52.6	84.6	64	Inoperable

12	12	809	1079	39.6	48	33.3	61	ERH

13	14	607	654	31.3	31.8	1.5	65	Inoperable

14	17	293	435	32.6	46.8	48.4	43	RH

15	20	383	453	31.8	38.5	18.2	55	RH

16	15	318	450	36	43.8	41.5	40	Inoperable

17	13	431	445	41.6	45.7	3.2	60	RH

18	14	358	438	29.5	36	22.3	46	RH

19	14	263	402	27.2	38.8	52.8	32	ERH

20	18	370	700	29	51.1	89.1	45	ERH

Avg.	14.3 ± 2.57	413.5 ± 133.0	556.3 ± 190.1	31.8 ± 6.0	41.3 ± 8.87	34.7 ± 23.7	52.7 ± 11.4	


CT: computed tomography; ERH: extended right hepatectomy; FLR: future liver remnant; GS: gelatin sponge; PVE: portal vein embolization; RH: right hepatectomy; SIBO: sheath injection with balloon occlusion; TLV: total liver volume.

Recanalization was confirmed in five patients, two of whom (Cases 2 and 8) required additional PVE because of the insufficient FLR/TLV ratio after initial PVE (20.1% and 28.3%, respectively). The additional PVE were performed within 4 weeks after the first: a second time PVE-SIBO with GS and transileocolic PVE with GS in Cases 2 and 8, respectively, and the FLR volume increased sufficiently. FLR increase ratios after the additional PVE were 17.9% and 58.6%.

The clinical outcomes are shown in ***Table 2***. Extended right hepatectomy or right hepatectomy were performed in 14 of the 20 patients after PVE-SIBO with GS. Three hepatic resections in Cases 3, 7, and 10 were cancelled because of peritoneal metastasis detected during the operation. The fourteen patients who had hepatectomy did not develop hepatic failure.

Treatments in Cases 11 and 13 were altered from surgery to chemotherapy due to progression of tumour invasion after PVE. Radiation therapy, not hepatic resection, was performed on the patient’s request in Case 16 after PVE.

## Discussion

Our retrospective study demonstrated a greater technical ease of PVE-SIBO with GS than those reported using other techniques and embolic agents to achieve an acceptable increase in FLR/TLV ratio prior to hepatic resection. Our procedure was simpler because it required no extra manipulations, such as in the reverse-curve catheter technique and cannulation to each branch of right portal vein. Few studies have discussed the operative time for PVE, which were defined with shorter ranges compared to ours [8]. Even though the average procedure time of 52.7 ± 11.4 minutes in this study can be clinically acceptable, it was not clear if PVE-SIBO with GS was superior to other techniques with respect to time because of lack of a common definition for procedure time.

Tsurusaki’s team achieved an adequate increase in FLR/TLV ratio utilizing the same method of PVE-SIBO, but they used absolute ethanol, a pure liquid, as their embolization agent [9]. Although easily injected through the gap between the sheath and catheter, absolute alcohol can induce strong hepatic inflammation after embolization that may cause adhesions that interfere with operational manipulation during hepatectomy [10,11]. Absolute ethanol, PVA and NBCA are nonabsorbable agents and reported to be effective in increasing the FLR/TLV ratio in PVE, but their use requires great skill and has a risk of non-target embolization [8,9,12,13,14]. The use of GS could be safer in cases of accidental contralateral embolization and in clinical situations in which the liver is not ultimately resected secondary to concomitant tumour progression after PVE. The cost of GS is lower than that of PVA or NBCA. Moreover, NBCA is not a suitable agent for PVE-SBIO technique due to the risk of adherence to the sheath and catheter. Although it is not covered by our national insurance system, ethylene vinyl alcohol may be a suitable non-adhesive embolic agent for PVE-SIBO.

We could achieve an increased ratio of FLR volume of 34.7 ± 23.7% using PVE-SIBO with GS. Tranchart and colleagues reported a slightly less increased FLR ratio (29.4 ± 6.9%) than ours using PVE with only GS [4], but this may be attributable to their not employing balloon occlusion, which was useful to fill the portal vein with a larger amount of GS. Other reports describe the performance of PVE using GS mixed with other embolic agents, including increase in FLR ratio of 30.7 ± 25.6% for PVE using GS mixed with coils and 30.0 ± 21.0% for PVE with GS mixed with EOI foam [15,16]. These procedures were more complex than ours and yielded inferior increase in FLR ratio compared to that of PVE-SIBO with GS alone.

The reported incidence of major complications, such as subcapsular hematoma, bile leakage, and non-targeted portal vein thrombosis after PVE, is 0–9% in the literature [12], but we observed no obvious complications, possibly because of the simplicity of our procedure.

Although we observed a relatively higher recanalization rate, 25.0%, than that reported by others, we consider this as acceptable because only two patients, 10.0% of our total cases, required additional embolization and were finally able to undergo hepatectomy. Furthermore, we observed a comparable recanalization rate to that of other reports of PVE with GS [4,14]. Recently, Berggren et al. reported almost an equivalent recanalization rate of 26.0% using PVE with NBCA [8].

All 14 patients who had hepatectomy did not develop hepatic failure. Although further investigation and accumulation of cases for PVE-SIBO are required, this hepatic failure rate after hepatectomy might be comparable to PVE with GS (1.50%), PVA or NBCA (4.0–10.0%) [8,17,18,19].

Our study was limited by its retrospective design, conducted in a single centre, and absence of randomization and comparison with other embolic agents. Further prospective, multi-institutional, randomized, larger patient population investigation utilizing PVE-SIBO with GS is required.

## Conclusion

We found percutaneous transhepatic PVE-SIBO with GS a safe and simple procedure with comparable efficacy to that of other previously reported techniques to increase the FLR volume prior to hepatic resection.
